# A deep learning feature importance test framework for integrating informative high-dimensional biomarkers to improve disease outcome prediction

**DOI:** 10.1093/bib/bbae709

**Published:** 2025-01-16

**Authors:** Baiming Zou, James G Xenakis, Meisheng Xiao, Apoena Ribeiro, Kimon Divaris, Di Wu, Fei Zou

**Affiliations:** Department of Biostatistics, University of North Carolina at Chapel Hill, Chapel Hill, NC 27599, United States; School of Nursing, University of North Carolina at Chapel Hill, Chapel Hill, NC 27599, United States; Department of Statistics, Harvard University, Cambridge, MA 02138, United States; Department of Biostatistics, University of North Carolina at Chapel Hill, Chapel Hill, NC 27599, United States; School of Dentistry, University of North Carolina at Chapel Hill, Chapel Hill, NC 27599, United States; School of Dentistry, University of North Carolina at Chapel Hill, Chapel Hill, NC 27599, United States; Department of Biostatistics, University of North Carolina at Chapel Hill, Chapel Hill, NC 27599, United States; School of Dentistry, University of North Carolina at Chapel Hill, Chapel Hill, NC 27599, United States; Department of Biostatistics, University of North Carolina at Chapel Hill, Chapel Hill, NC 27599, United States; Department of Genetics, University of North Carolina at Chapel Hill, Chapel Hill, NC 27599, United States

**Keywords:** complex association, dimension reduction, interpretable and scalable predictive modeling, non-parametric feature selection, stable deep neural network

## Abstract

Many human diseases result from a complex interplay of behavioral, clinical, and molecular factors. Integrating low-dimensional behavioral and clinical features with high-dimensional molecular profiles can significantly improve disease outcome prediction and diagnosis. However, while some biomarkers are crucial, many lack informative value. To enhance prediction accuracy and understand disease mechanisms, it is essential to integrate relevant features and identify key biomarkers, separating meaningful data from noise and modeling complex associations. To address these challenges, we introduce the High-dimensional Feature Importance Test (HdFIT) framework for machine learning models. HdFIT includes a feature screening step for dimension reduction and leverages machine learning to model complex associations between biomarkers and disease outcomes. It robustly evaluates each feature’s impact. Extensive Monte Carlo experiments and a real microbiome study demonstrate HdFIT’s efficacy, especially when integrated with advanced models like deep neural networks. Our framework shows significant improvements in identifying crucial features and enhancing prediction accuracy, even in high-dimensional settings.

## Introduction

A growing body of research has highlighted that many human diseases’ onset and severity are influenced by a combination of behavioral factors, clinical features, and molecular biomarkers [1–3]. For instance, the complex oral disease, e.g. early childhood caries (ECC), involves interactions between oral microbiome signatures and other behavioral features in addition to clinical factors [4–8]. Imbalances in the oral microbiome, disrupting the delicate microbial balance, have been linked to various oral and systemic diseases, such as periodontitis [9–13]. Furthermore, an imbalanced oral microbiome has been associated with systemic diseases like diabetes and cardiovascular disease [14, 15]. Molecular biomarkers have been extensively studied for diagnosing and understanding the development of complex human diseases, offering potential for developing new diagnostic and therapeutic strategies [16–20]. Alongside molecular biomarkers, behavioral and clinical factors, as well as dietary habits, significantly impact human health. Factors such as tobacco use, alcohol consumption, and dietary habits like the consumption of sugary or acidic snacks and drinks are associated with increased risks of oral cancer, periodontitis, and caries [21, 22]. The combined effects of these behavioral and clinical factors can have negative consequences on health [23, 24].

Integrating behavioral and clinical features with molecular profiles is therefore crucial for improving human disease diagnosis and outcome predictions. Furthermore, this integration provides a comprehensive understanding of the complex interplay between genetic factors, environmental exposures, and individual health outcomes. However, these factors may interact through intricate pathways influencing disease development and severity [25, 26]. Additionally, the number of molecular markers often surpasses the available sample size, resulting in the curse of dimensionality. Moreover, while some biomarkers are strongly associated with disease outcomes, the majority are not informative and act as nuisance variables. As such, merely combining all features and biomarkers in predictive models does not necessarily improve prediction power. Therefore, it is critical to differentiate important biomarkers from nuisance variables and integrate them with critical behavioral and clinical features, facilitating more accurate disease outcome predictions and shedding light on disease development mechanisms. However, this presents several major challenges: (1) how to robustly model the complex association between disease outcomes and predicting features, (2) how to construct appropriate test statistics that enable robust statistical inference for evaluating the contribution of each feature under the complex functional relationships, and (3) how to address the curse of dimensionality. Conventional analytic methods, which rely on strong linear additive assumptions, are often inadequate in this context. Fortunately, many machine learning methods have been developed to robustly model these complex associations and have been adopted for integrating various high-dimensional (e.g. omics) data [1–3, 27–29].

Herein, we investigate several commonly employed machine learning models regarding their performances under high-dimensional settings, particularly deep neural networks (DNN), which excel in approximating complex associations between predicting features and disease outcomes. In particular, for the DNN method, we adopt a recently proposed stable DNN ensemble by employing a scoring algorithm that enhances the prediction stability over the conventional DNN due to the random parameter initialization [30]. While machine learning methods are powerful for modeling complex associations, their abstract nature often hampers the interpretation of each feature’s contribution to disease phenotypes. To address this limitation, we employ a newly developed permutation-based feature importance test (PermFIT) [31] for machine learning models to robustly identify important features under different association complexities. However, the PermFIT framework may not effectively identify important markers under high-dimensional settings where machine learning models may be inadequately trained. Importantly, machine learning models become computationally intractable in very high-dimensional settings. Therefore, we first conduct feature pre-screening for dimension reduction to create a targeted compact feature space, enabling the PermFIT framework to more effectively identify important features (referred to as HdFIT). We apply the HdFIT framework to different machine learning models to analyse data from various data complication settings and an oral microbiome study, aiming to identify important biomarkers associated with oral disease outcomes in a cohort of young children [32]. The paper proceeds as follows: Section 2 describes the revised stable DNN ensemble method and the HdFIT procedure, while the Results section conducts extensive numerical studies to investigate the proposed HdFIT framework performance under different data complication settings and applies the HdFIT-based procedure to predict disease outcomes and identify important clinical factors and molecular biomarkers in a recent oral microbiome study data set, drawing comparisons among different machine learning models. The paper concludes with a brief discussion.

## Method

### Machine learning methods for modeling complex associations

In this study, we focus on a continuous disease outcome $Y$ (e.g. a personal level quantitative trait representing total caries/cavities experienced) using an observed $p$-dimensional set of features $\mathbf{X} = (X_{1},..., X_{p})$, which includes behavioral factors (e.g. sweetened snack & beverage consumption), demographic features (e.g. age, gender) and molecular biomarker profiles. Conditional on the observed features $\mathbf{X}$, our primary interest is to build predictive models to estimate the conditional expectation for a continuous outcome $Y$, which is represented as $\xi (\mathbf{X}) \equiv \text{E}(Y|\mathbf{X})$, such that the outcomes for future patients can be accurately and robustly predicted. Traditionally, this quantity can be estimated via a multiple linear regression model. However, the parametric modeling strategy require strong linear additive assumption on the relationship between the observed features $\mathbf{X}$ and the disease outcomes $Y$—assumptions that may be tenuous and, in practice, unverifiable. To relax the restrictive linear additive assumptions, machine learning methods are often adopted. Herein, we investigate two frequently used machine learning models for their performance in approximating the unspecified function $\xi (\mathbf{X})$: (1) a non-parametric tree-based algorithm using parallel tree growing with subsamples, i.e. Random Forest (RF) [33, 34] and (2) a non-parametric fully connected DNN [35, 36] in addition to a regularized regression, i.e. least absolute shrinkage and selection operator (Lasso) [37, 38]. Furthermore, we adopt the following procedures to improve the stability of DNN method.

### Procedures for establishing stable DNN ensemble

To address the potential unstable predictions of the conventional DNN algorithm, we adopt the following stability strengthening procedures. First, we adopt bootstrap aggregating—a machine learning ensemble meta-algorithm [39] to increase the stability and accuracy of a single DNN [40]. However, this still does not guarantee the stable prediction of the final ensemble model, in particular when the dimensionality is not particularly low (i.e. $p\approx n$) due to the random parameter initialization in the DNN algorithm. To further boost the DNN performance, we adopt a filtering algorithm to exclude poorly performing bagged DNN models. That is, we filter out those poorly performing DNNs from the final ensemble according to the following criteria. For the $k^{\text{th}}$ bootstrap sample, a performance score, $r_{k}$, is computed as


\begin{align*}& r_{k} = \frac{1}{|D_{{O_{k}}}|}\sum_{i\in \mathcal{D}_{{O_k}}} \left\{(y_{i} - \overline{y}_{O_{k}})^{2} - (y_{i} - \widehat{y}_{i{k}})^{2}\right\} \end{align*}

where $D_{O_{k}}$ is the set of out-of-bag samples with associated cardinality $|D_{{O_{k}}}|$, $\overline{y}_{O_{k}} = \sum _{i\in D_{O_{k}}}y_{i}/|D_{O_{k}}| $ and $\widehat{y}_{ik} = \widehat{f}_{k}(\boldsymbol{{x}_{i}})$ ($i\in D_{O_{k}}$). $(y_{i} - \widehat{y}_{i{k}})^{2}$ is included in $r_{k}$ evaluation to deal with the potential outlier observations.

To determine the optimal number of DNN models to be retained in the final ensemble, we rank the models based on their performance scores, denoting these ranked scores as $r_{(1)}\ge \cdots \ge r_{(K)}$. Aggregating the top $q$ DNNs, the prediction for $\boldsymbol{x}_{i}$$(i = 1,\cdots ,n)$ is given by


\begin{align*}& \widehat{f}^{(q)}(\boldsymbol{x}_{i}) = \frac{1}{q}\sum_{k\le q}\widehat{f}^{(k)}(\boldsymbol{x}_{i}),\ (q = 1,...,K) \end{align*}

where $\ \widehat{f}^{(k)}(\cdot )$ is the fitted DNN corresponding to performance score $r_{(k)}$. The optimal number of DNNs utilized by the ensemble, $q_{\text{opt}}$, is determined by minimizing the training loss, such that


\begin{align*}& q_{\text{opt}} = \mathop{\text{argmin}}\limits_{1\le q\le K} \sum_{i=1}^{n} \ell\{\widehat{f}^{(q)}(\boldsymbol{x}_{i}), y_{i}\} \end{align*}

based on which we obtain the revised bagging prediction $\widehat{f}^{(q_{\text{opt}})}(\boldsymbol{x})$ for a new observation with input profile $\boldsymbol{X}=\boldsymbol{x}$.

### PermFIT for low-dimensional data

Although machine learning methods can relax restrictive assumptions made in the traditional parametric approach and improve prediction accuracy, they lack transparency to interpret the contribution of each feature on the disease outcome. To this end, we can evaluate the role of each feature using rigorous statistical inference for machine learning models by adopting a PermFIT procedure [31], as described briefly below. Based on the PermFIT, we derive a powerful framework to identify important clinical features and molecular biomarkers to improve disease prediction accuracy.

We define the importance score of feature $X_{j}$ (i.e. the $j^{\text{th}}$ feature in $\mathbf{X}$$(j=1,...,p)$) as the expected squared difference between $\xi (\mathbf{X})$ and $\xi \left (\mathbf{X}^{(j)}\right )$, where $\mathbf{X}^{(j)} = (X_{1},..., X_{j-1}, X_{j^{\prime}}, X_{j+1},..., X_{p})$ is a rearranged version of $\mathbf{X}$ with its $j^{th}$ feature replaced by $X_{j^{\prime}}$, a random permutation of the elements of $X_{j}$. The importance score $\Lambda _{j}$ can be re-expressed as $\Lambda _{j} = \operatorname{E}_{\mathbf{X}, \mathbf{X}_{j^{\prime}}}[\xi (\mathbf{X})-\xi \left (\mathbf{X}^{(j)}\right )]^{2}$, which is zero only when $\xi (\mathbf{X})\equiv \xi (\mathbf{X}^{(j)})$, implying no contribution of $ X_{j^{\prime}}$ on $\xi (\mathbf{X})$ conditional on the other covariates. The stronger the impact of $ X_{j^{\prime}}$ on $\xi (\mathbf{X})$, the larger $\Lambda _{j}$ is expected to be. Furthermore, $\Lambda _{j}$ can be estimated empirically. Let $X_{j}^{\prime} = (X_{s_{1},j},..., X_{s_{n},j})$ be a random sample of the elements in $X_{j}$ without replacement, and the empirical permutation importance score be $\Lambda _{j}^{(P)} = \frac{1}{n}\sum _{i=1}^{n} \Lambda _{ij}^{(P)}$, where $ \Lambda _{ij}^{(P)} = \{Y_{i} - \xi (\mathbf{X}_{i\cdot }^{(j)})\}^{2} - \{Y_{i} - \xi (\mathbf{X}_{i\cdot })\}^{2} $ with $\mathbf{X}_{i\cdot } = (X_{i1},..., X_{ip})$ and $\mathbf{X}_{i\cdot }^{(j)} = (X_{i1}, \cdots , X_{i,j-1}, X_{s_{i},j},$$X_{i,j+1},\cdots , X_{ip})$. Note that $\operatorname{E}[\Lambda _{j}^{(P)}] = \operatorname{E}[\Lambda _{ij}^{(P)}] = \frac{n-1}{n} \Lambda _{j}$. The estimation of $\xi (\cdot )$, i.e. $\widehat \xi (\cdot )$, can be obtained using different machine learning models when the dimension is not high or Lasso regression regardless if the dimension is high or not. Here, we consider the following two frequently used machine learning models: RF and DNN (the particular DNN method we used is the stable DNN ensemble described above). We then estimate $\Lambda _{j}^{P}$ as


\begin{align*} & \widehat{\Lambda}_j^{(P)} = \frac{1}{n}\sum_{i=1}^{n} [\{Y_i - \widehat\xi(\textbf{X}_{i\cdot}^{(j)})\}^2 - \{Y_i - \widehat\xi(\textbf{X}_{i\cdot})\}^2] \end{align*}

To avoid potential overfitting of $\widehat \xi (\cdot )$, we employ a cross-fitting strategy to separate the input data into training and validation sets, with the training set used for generating $\widehat \xi (\cdot )$ and the validation set for estimating $\widehat{\Lambda }_{j}^{(P)}$. Letting $\widehat \xi _{T}(\cdot )$ be the estimate of $\xi (\cdot )$ from the training set, and $\mathcal{D}_{V} = \{Y_{i}, \mathbf{X}_{i\cdot }\}_{i=1}^{n_{V}}$ be the validation set, we obtain the feature importance score estimate $\widehat{\Lambda }_{j}^{(P)}$ and the associated estimate of its variance as


(1) \begin{align*} \widehat{\Lambda}_{j}^{(P)} &= \frac{1}{n_{V}}\sum_{i=1}^{n_{V}} \widehat{\Lambda}_{ij}^{(P)} \notag \\ &= \frac{1}{n_{V}}\sum_{i=1}^{n_{V}} \left[\{Y_{i} - \widehat\xi_{T} (\mathbf{X}_{i\cdot}^{(j)})\}^{2} - \{Y_{i} - \widehat\xi_{T}(\mathbf{X}_{i\cdot})\}^{2}\right] \end{align*}

(2) \begin{align*} \,\,\,\widehat{Var}[\widehat{\Lambda}_{j}^{(P)}] &= \frac{1}{n_{V}}\sum_{i=1}^{n_{V}} \left[ \widehat{\Lambda}_{ij}^{(P)} - \widehat{\Lambda}_{j}^{(P)} \right]^{2} .\qquad\qquad\qquad\qquad\qquad\ \ \, \end{align*}

We can then construct a test statistic for the feature importance hypothesis test of feature $X_{j}$ as


\begin{align*}& \lambda=\frac{\widehat{\Lambda}_{j}^{(P)}}{\sqrt{\widehat{\text{Var}}[\widehat{\Lambda}_{j}^{(P)}]}} \end{align*}

### Feature screening for identifying informative high-dimensional biomarkers

When integrating genetics or microbiome profiles, the number of molecular biomarker often exceeds or substantially surpasses the available sample size. Under the high-dimensional settings, machine learning models may not be well trained, thus the PermFIT framework using machine learning methods may not identify important biomarkers effectively, leading to poor prediction performance. Under very high-dimensional settings, machine learning methods will become computationally intractable. Therefore, to achieve robust important feature identification under high or very high-dimension settings, it is critical to conduct computationally efficient dimension reduction before adopting the machine learning methods for robust feature importance identification and outcome predictions. Herein, we present two convenient yet effective feature screening methods for dimension reduction. For gene expression data, usually highly expressed genes could have more impacts on outcomes. Therefore, we may adopt an unsupervised learning method. We define the informative score for a biomarker $Z_{k}$ as the mean of the log-transformed biomarker measurements, i.e. $\delta _{k}=\frac{1}{n}\sum _{i=1}^{n}(\text{log}(Z_{(k,i)})$, where $n$ is the sample size and $Z_{(k,i)}$ refers to the $k^{th}$ biomarker from sample $i$. We employ the log transformation due to the typically very skewed nature of gene expression measurement distributions and extremely large numerical values. This informative score can be equivalently regarded as a geometric mean of the biomarker measurements. Intuitively, the magnitude of this value is directly associated with how informative a biomarker might be. Only a pre-specified number of biomarkers (those with the top informative scores) are then integrated with the other low-dimensional behavioral and clinical features by using the aforementioned PermFIT framework to evaluate each individual feature’s impact. Alternatively, we can employ supervised learning to achieve dimension reduction. A straightforward approach to supervised learning involves evaluating the correlation between the outcome variable, denoted as $Y$, and each of the high-dimensional features, represented by $Z_{k}$, using the training samples. This can be expressed as $\delta _{k} = \text{corr}(Y, Z_{k})$. The top $K$ pre-specified features with the highest absolute values of $\delta _{k}$ will be retained. A more robust method of supervised learning is to perform multiple linear regression while adjusting for potential confounding factors. Specifically, for each high-dimensional feature, denoted as $Z_{k}$, we can fit a multiple linear regression model as follows:


\begin{align*}& Y_{i} = \alpha_{0} + \alpha_{Z^{\ast}_{k}} Z^{\ast}_{i,k} + \boldsymbol{\alpha}_{\mathbf{X}}\mathbf{X}_{i} + \boldsymbol{\alpha}_{(\mathbf{X},Z^{\ast}_{k})}\mathbf{X}_{i} * Z^{\ast}_{i,k} + \epsilon \end{align*}

where $i=1,\cdots , m$ with $m$ being the number of training samples. $Z^{\ast }_{i,k}=\text{log}({Z_{i,k}})$ or the normalized high-dimension biomarker measurements. $\mathbf{X}$ are the low-dimensional behavioral, clinical and demographic features being adjusted as confounding variables, and $\epsilon $ is the random error term. We obtain the test statistic for each high-dimension feature $Z_{k}$ as


(3) \begin{align*}& \delta_{k} = \frac{|\hat{\alpha}_{Z^{\ast}_{k}}|}{\widehat{\text{SE}}(\hat{\alpha}_{Z^{\ast}_{k}})} \end{align*}

**Figure 1:**
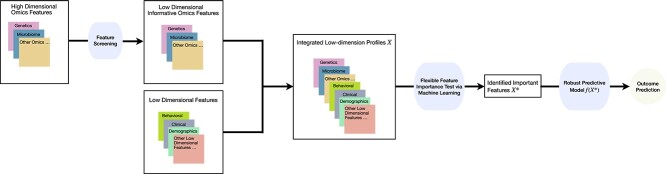
HdFIT framework.

The first $K$ pre-specified high-dimension features with the largest $\delta _{k}$ are then kept to be integrated with the low-dimension features $\mathbf{X}$ using the PermFIT framework. In addition to the above linear regression model by adjusting for the linear terms of behavorial, clinical and demographic factors plus the mutual interactions between these low-dimension variables and each high-dimension feature, more complex nonlinear (e.g. quadratic) terms can be incorporated into the regression model for feature screening of high-dimension features. The permutation-based High-dimensional Feature Importance Test (HdFIT) framework is summarized in Fig. 1.

## Results

### Simulation studies

To explore the applicability and evaluate the performance of the proposed HdFIT-DNN feature importance identification for predictive modeling under high-dimension settings, we conducted numerical studies under two scenarios that captured different type of data structure complexity. For each of these scenarios, we allowed the molecular feature matrix, $\boldsymbol{Z}$, to be comprised of high, medium high, and very high dimension. A subset of high-dimension features of size $10$ each following a normal distribution as $N(0,1)$ was assumed to affect disease outcomes. Besides the molecular biomarkers, a set of $20$ behavioral and clinical features $\mathbf{X}\sim N(0,1)$ are collected but only $10$ of these low-dimension features have impact on the disease outcomes $Y$ as follows:

**Scenario 1 (Linear association)**: in this scenario, we considered a simple data structure where the sets of covariates influence the disease outcome $y$ via a model that contain only linear terms such that


\begin{align*}& y = \alpha_{0} + \sum_{q=1}^{10} \alpha_{(z,q)} z_{q} + \sum_{q=1}^{10} \alpha_{(x,q)} x_{q} + \epsilon \end{align*}

**Scenario 2 (Nonlinear association)**: building on Scenario 1, we increased the data complexity by adding the quadratic terms and interactions of the linear terms, as follows:


\begin{align*}y &=\alpha_{0} + \sum_{q=1}^{10}\alpha_{(z,q)} z_{q} + \sum_{q=1}^{10} \alpha_{(x,q)} x_{q} + \sum_{q=1}^{10} \alpha_{(qua,z,q)} z_{q}^{2} \\ &\quad +\sum_{q=1}^{10} \alpha_{(qua,x,q)} x_{q}^{2} + \sum_{q=1}^{10} \alpha_{(int,q)} x_{q}z_{q} + \epsilon \end{align*}

The random error term $\epsilon $ is assumed to follow $N(0,1)$ distribution. Further, for each scenario, we ran three settings that differed in the number of nuisance high-dimensional covariates generated. In the first setting, we generated $470$ additional continuous nuisance covariates (following normal distribution $\sim N(0,1)$), which have no effect on the disease outcome. In the second and third setting, we generated $970$ and $9970$ such nuisance covariates, respectively. Thus, this resulted in either $p$=500, 1000, or 10 000 observed covariates in total. The parameters $\alpha _{0}$, $\alpha _{(z,1)}, \alpha _{(x,1)}, \cdots , \alpha _{(z,10)}, \alpha _{(x,10)}$, $\alpha _{(qua,z,1)},\alpha _{(qua,x,1)},\cdots ,\alpha _{(qua,z,10)},\alpha _{(qua,x,10)}$, and $\alpha _{(int,1)}, \cdots ,\alpha _{(int,10)}$ are drawn from standard normal distribution at the beginning of each simulation study and remain unchanged in the subsequent $1000$ Monte Carlo replications.

Under the simulation settings described above, with a sample size of $n=500$, we compared the performance of the stable DNN model we adopted against the traditional DNN model (tDNN) in addition to two other machine learning methods: RF and Lasso. In each Monte Carlo replication, the generated data was split into two parts, reserving $50$ samples for the testing set, and using the remaining samples for feature screening and model training. For the stable DNN method, we configured the model with four hidden layers, each containing $50, 40, 30$, and $20$ hidden nodes (from the first to the last layer), using the rectified linear unit as the activation function. In the RF method, we grew 1000 trees, and all other tuning parameters were determined through cross-validation. The tuning parameters used in Lasso method were determined using cross-validation approach. Additionally, in the PermFIT framework, we set the p-value cutoff to $0.1$ to identify the important features for inclusion in the DNN and RF predictive models, known as PermFIT-DNN and PermFIT-RF, respectively. In the HdFIT framework, we first conducted feature screening to select the top $80$ features with the largest test statistics (3) using a multiple linear regression by adjusting for the linear terms of $20$ low-dimension features regardless if they have impacts on the outcome or not. Subsequently, we applied PermFIT-DNN and PermFIT-RF to identify the important features along with $20$ behavioral and clinical features from total $100$ input features. The identified important features are then included in the DNN and RF predictive models, referred to as HdFIT-DNN and HdFIT-RF, accordingly. To demonstrate the efficiency gain from the feature screening, we also included randomly screened 80 high-dimensional features along with 20 low-dimensional features being passed to the downstream feature importance framework PermFIT to identify the important features using machine learning methods RF and stable DNN, and denoted as Control-RF and Control-DNN, respectively. Performance comparisons were conducted using two metrics: the mean squared prediction error (MSPE)—a quantitative measure of the average squared difference between the predicted disease outcomes and actual observed disease outcomes, defined as $\frac{1}{m}\sum _{i=1}^{m}(y_{i}-\hat{y}_{i})^{2}$, where $m$ represents the testing sample size, and $\hat{y}_{i}=\hat{\xi }_{i}(\mathbf{x})$ denotes the predicted outcome for testing sample $i$ using the corresponding machine learning model or Lasso method. Additionally, we used the Pearson correlation coefficient (PCC)—measuring the strength and direction of the linear relationship between predicted and actual observed outcomes, as a performance metric. The results for Scenario $1$ and $2$ under three high-dimensional settings are presented in Tables 1 and 2, respectively.

**Table 1:** Performance comparisons with simple linear effects (Scenario 1)

Method	$p$ =500		$p$ =1000		$p$ =10 000	
	**MSPE**	**PCC**	**MSPE**	**PCC**	**MSPE**	**PCC**
Lasso	1.300	0.988	1.362	0.988	1.610	0.987
RF	28.73	0.754	30.25	0.732	NA	NA
PermFIT-RF	23.29	0.826	25.42	0.799	NA	NA
**Control-RF**	40.76	0.417	43.65	0.348	44.65	0.315
HdFIT-RF	21.14	0.852	22.01	0.842	24.69	0.812
**tDNN**	2.966	0.971	3.500	0.968	NA	NA
DNN	2.306	0.980	3.240	0.976	NA	NA
PermFIT-DNN	1.878	0.982	2.188	0.980	NA	NA
**Control-DNN**	39.51	0.449	42.90	0.378	43.88	0.342
HdFIT-DNN	1.663	0.984	1.744	0.983	2.513	0.976

NA: computationally intractable.

**Table 2:** Performance comparisons with complex nonlinear effects (Scenario 2)

Method	$p$ =500		$p$ =1000		$p$ =10 000	
	**MSPE**	**PCC**	**MSPE**	**PCC**	**MSPE**	**PCC**
Lasso	71.30	0.715	72.97	0.715	90.24	0.634
RF	103.0	0.623	108.0	0.585	NA	NA
PermFIT-RF	87.75	0.711	94.44	0.668	NA	NA
**Control-RF**	94.08	0.563	97.08	0.536	94.00	0.556
HdFIT-RF	82.20	0.744	85.13	0.728	93.35	0.670
**tDNN**	81.52	0.610	96.64	0.515	NA	NA
DNN	72.30	0.684	86.00	0.615	NA	NA
PermFIT-DNN	21.58	0.921	35.72	0.861	NA	NA
**Control-DNN**	86.73	0.578	92.57	0.541	91.08	0.550
HdFIT-DNN	17.67	0.935	20.72	0.923	41.89	0.829

NA: computationally intractable.

The results presented in Table 1 highlight the efficacy of the stable DNN compared with the traditional DNN in modeling the linear associations between risk factors and disease outcomes. For instance, the predicted MSPE from the stable DNN is over $22\%$ smaller than that from the tDNN. This efficacy gain is due to the fact that the stable DNN adopted a revised ensemble algorithm by filtering out poorly performing bootstrapped DNN models. However, it is noteworthy that machine learning methods, including tDNN, DNN, and RF, become computationally intractable when dealing with very high-dimensional data. In contrast, the Lasso method demonstrates its effectiveness in modeling linear associations across a wide range of dimensionalities.

It is important to observe that as the dimensionality of the data increases, all methods experience diminishing effectiveness in accurately modeling the association relationships, resulting in a noticeable decline in prediction accuracy. For instance, when the dimensionality ($p$) increases from $500$ to $1000$, the MSPE for the RF method increases from $28.73$ to $30.25$, the DNN method’s MSPE rises from $2.306$ to $3.240$, and the Lasso method’s MSPE increases from $1.300$ to $1.362$. A direct comparison between the stable DNN and RF methods underscores the clear advantage of the DNN method in effectively modeling linear association relationships, leading to significantly improved prediction accuracy. For instance, with a dimensionality of $500$, the MSPE is $28.73$ for RF compared to $2.306$ for the stable DNN, representing a more than 12-fold difference in predictive performance.

The results in Table 1 also demonstrate that feature selection is beneficial in identifying important features to be included in the predictive model, significantly improving outcome prediction regardless of the machine learning method used. For example, with $p$=1000, conducting feature selection under the PermFIT framework using the DNN method (i.e. DNN vs PermFIT-DNN) improves the MSPE from $3.240$ to $2.188$, representing an approximately $48.0\%$ improvement. Additionally, the proposed HdFIT framework allows for more effective feature selection within a narrowed-down search space, resulting in further significant improvement in outcome prediction. For instance, with $p$=1000, the MSPE for PermFIT-DNN and HdFIT-DNN is $2.188$ and $1.744$, respectively, representing another $25.5\%$ improvement. Notably, the efficiency gain of HdFIT is due to the informative features are screened and kept in the downstream feature selection. This can be clearly observed when comparing Control-DNN vs HdFIT-DNN or Control-RF vs HdFIT-RF. An another advantage of the HdFIT framework is that it enables machine learning methods to be tractable under very high-dimensional settings. Furthermore, the MSPE based on the HdFIT framework remains relatively stable regardless of whether the dimension is high or very high and irrespective of the machine learning method used. In addition to these observations, we noticed that the Lasso method and the HdFIT-DNN method have comparable predicted MSPE when the dimension is high and median-high, while the predicted PCC from the Lasso method is almost identical to that from the HdFIT-DNN method regardless if the dimension is high or very high.

The results in Table 2 reaffirm the observation of larger MSPE with higher dimension across all methods (Lasso, RF, DNN) under the nonlinear and non-additive setting. Additionally, within each machine learning method, feature selection proves helpful in improving outcome prediction accuracy. Notably, feature screening allows for a significantly narrowed-down search space, resulting in more effective identification of important features and remarkable improvements in outcome prediction accuracy. However, a remarkable difference between the results of Tables 1 and 2 becomes evident. Specifically, the MSPE from the Lasso method is no longer comparable to that from the HdFIT-DNN method under high and median-high dimension settings with nonlinear and non-additive effects. Instead, the predicted MSPE via the Lasso method is substantially larger than that from the HdFIT-DNN method. For instance, with $p=500$, the predicted MSPE via the Lasso method and HdFIT-DNN method are $71.30$ and $17.67$, respectively, representing a more than $300\%$ difference. Similar observations can be noticed for PCC prediction, i.e. significant larger predicted PCC from HdFIT-DNN ($0.935$) than that from Lasso ($0.715$). As the dimension increases to very high, HdFIT-DNN still remarkably outperforms Lasso for MSPE and PCC predictions, i.e. $(41.89,0.829)$ vs $(90.24, 0.634)$. These findings clearly indicate that the Lasso method has limited capability in modeling the complex association between risk features and disease outcomes, resulting in poor prediction accuracy. On the other hand, our proposed HdFIT-DNN framework effectively identifies important features and remarkably improves outcome prediction accuracy, regardless of whether the feature space dimension is high or very high, and regardless of the complexity of association. Most importantly, the HdFIT-DNN is scalable to deal with very high dimension data for robustly identifying important features to improve outcome prediction accuracy, indicating its usefulness in clinical practice.

### Real application for integrating informative high-dimensional biomarkers

To demonstrate the practical application of our proposed HdFIT-DNN framework, we conducted a study focusing on integrating behavioral and demographic features with oral microbiome profiles from a cohort of young children. The objective was to identify important factors that could enhance the prediction of oral disease outcomes [32]. The cohort comprised $288$ samples, each with $9$ recorded behavioral factors and demographic features. Additionally, the samples were analysed for abundance of $417$ microbiome species measured in WGS shotgun DNA sequencing, quantified using the Bracken method, which utilizes taxonomic assignments and genome information to estimate abundance at the species level [41]. The collected disease outcomes included a continuous person-level quantitative trait representing total caries lesions, with a mean value of $13.69$ and a standard deviation of $15.18$. Table 3 presents the summary statistics for the behavioral and demographic factors obtained from the study.

**Table 3:** Summary statistics for behavioral factors and demographic features

Categorical feature		**# of Samples**	**Percentage**
Gender	Boy	149	51.74
	Girl	139	48.26
Race	White	102	35.42
	Black	112	38.89
	Other	74	25.69
$\ge 2$ sweetened snacks&beverages/day	Yes	189	65.63
	No	99	34.37
Bed with anything other than water	Yes	70	24.31
	No	218	75.69
Lives with optimally fluoridated water	Yes	124	43.06
	No	164	56.94
Twice a day tooth brushing	Yes	184	63.89
	No	104	36.11
Regular dentist for cleanings/check-ups	Yes	238	82.64
	No	50	17.36
**Continuous feature**		**Mean**	**SD**
Age (in month)		52.23	7.79

In our real application study, we also evaluated the predictive performance of two machine learning models as we investigated in the simulation experiment, namely RF and stable DNN, under two distinct scenarios with varying numbers of potential predictors for a continuous person-level quantitative trait, i.e. Scenario 1: Clinical Only—utilizing only the nine behavioral/demographic features as predictors; Scenario 2: Clinical+DNA—combining the nine behavioral/demographic features with all microbiome DNA biomarkers as predictors. For evaluation, we employed an approximate $10:1$ cross-validation approach, where $25$ samples were randomly selected for testing, while the remaining $263$ samples were used for model training. DNN architecture used and other hyper-parameters determination strategy for DNN, RF, and Lasso are the same as in simulation study.

**Figure 2:**
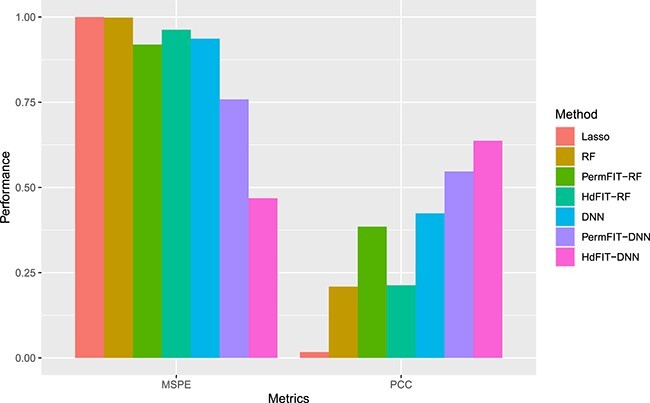
Prediction comparisons among machine learning models with important feature identification.

In Scenario 2 (Clinical+DNA) which corresponds to median-high dimension setting, we further expanded our analysis using the PermFIT framework and Lasso. A $10$-fold cross-validation and $200$ permutations were employed to identify important features from total $426$ features using machine learning methods, i.e. RF and the stable DNN, for predicting the continuous trait in the testing samples. The identification of these important features was performed in the learning samples using a significance level of $p=0.1$. We designated these modified scenarios as PermFIT-RF and PermFIT-DNN. While the PermFIT framework enhances the machine learning models’ capabilities to not only predict outcomes but also identify important features in high-dimensional scenarios (i.e. Clinical+DNA), it does not necessarily achieve the optimal performance. To address this, we incorporated an additional feature screening step, retaining the top $100$ DNA biomarkers with the highest impact scores evaluated via the supervised learning scheme, i.e. (3), by adjusting for behavior and clinical factors in addition to the interactions of these features with respect to each DNA biomarker against the training samples. Subsequently, we applied the PermFIT procedure for important feature identification from the total $109$ input features on the training samples using the stable DNN (HdFIT-DNN) and RF (HdFIT-RF) methods. Results are presented in Table 4 and a selection displayed visually in Fig. 2.

**Table 4:** Outcome predictions via identified important features

**Method**	Clinical Only		Clinical + DNA	
	**MSPE**	**PCC**	**MSPE**	**PCC**
Lasso	NA	NA	85.00	0.017
RF	104.25	0.112	84.88	0.209
PermFIT-RF	NA	NA	78.13	0.385
HdFIT-RF	NA	NA	81.82	0.213
DNN	76.33	0.285	79.60	0.424
PermFIT-DNN	NA	NA	64.48	0.547
HdFIT-DNN	NA	NA	39.83	0.637

NA: no feature selection under low-dimension.

To enable effective comparisons in Fig. 2, we scaled and normalized the MSPE by that of the Lasso method in the Clinical+DNA setting. Specifically, the MSPE for the Clinical+DNA scenario (which was $85.00$, as shown in Table 4) was set to 1.00. The results in Table 4 demonstrate that the Lasso method has the largest MSPE yet the smallest predicted PCC among all three methods considered (i.e. Lasso, RF, and DNN). This could be intuitively interpreted as the existence of nonlinear effects on the disease outcomes due to the clinical features and DNA biomarkers that the Lasso method could fail to model appropriately. Results of Table 4 further highlight that using only clinical features as predictors in the machine learning models results in limited prediction power in terms of both MSPE and PCC. Conversely, incorporating DNA biomarkers improves the performance, especially evident when examining the outcomes of the stable DNN method, as depicted in Fig. 2. This visualization demonstrates a clear pattern of improvement in predicted MSPE and PCC as we consider successive scenarios: (1) using clinical/demographic features in combination with the $417$ DNA biomarkers (Clinical+DNA) in the DNN; (2) identifying important features among the combined clinical features and DNA profiles using the PermFIT framework (PermFIT-DNN); and (3) identifying important features from among the combined clinical features and pre-screened DNA biomarkers using the HdFIT framework (HdFIT-DNN). Specifically, for the stable DNN model, the predicted PCC using only the nine clinical features is $0.285$. When the $417$ DNA biomarkers are combined with these clinical features, the predicted PCC becomes $0.424$, representing a nearly $49\%$ improvement.

Furthermore, when the PermFIT framework is added to the stable DNN method (i.e. PermFIT-DNN) to identify important features from the combined total of $425$ clinical features and DNA biomarkers, $43$ important features are identified, including $4$ clinical and demographic features, i.e. ‘lives with optimally fluoridated water’, ‘more than $2$ sweetened snacks&beverages/day’, ‘bed with anything other than water’, and ‘age’, and $39$ molecular biomarkers. With the identified important features and biomarkers included in the stable DNN predictive model, the predicted MSPE decreases from $79.60$ to $64.48$, a nearly $23\%$ improvement, and the PCC increases from $0.424$ to $0.547$, representing an approximate $29\%$ improvement compared with the corresponding prediction results from the stable DNN model with all $425$ clinical features and DNA biomarkers included. This clearly demonstrates that the stable DNN not only robustly models the complex associations between clinical features, biomarkers, and disease outcomes but also effectively identifies the important features associated with the disease outcome by incorporating the PermFIT framework, leading to significantly improved prediction accuracy. In addition to the two scenarios, we also conducted analysis including only DNA biomarkers using Lasso, RF, PermFIT-RF, DNN, and PermFIT-DNN with the corresponding (MSPE, PCC) as follows: (76.89, 0.051), (90.38, 0.173), (70.53, 0.424), (100.7, 0.334), and (104.0, 0.091) accordingly. This result clearly suggests that using clinical features only or microbiome biomarkers only can not appropriately model oral disease outcomes and lead to poor predictions no matter which machine learning model is adopted. Instead, the clinical features and microbiome biomarkers should be integrated for modeling oral diseases.

By adding the screening step to pre-screen $100$ DNA biomarkers from $417$ markers in addition to $9$ clinical and demographic features to identify important features using the stable DNN method under the PermFIT framework (i.e. transitioning from PermFIT to HdFIT framework), i.e. HdFIT-DNN, a total of $19$ important features are identified. Among these identified important features, $4$ are clinical and demographic features, i.e. ‘more than $2$ sweetened snacks&beverages/day’, ‘lives with optimally fluoridated water’, ‘regular dentist for cleanings/check-ups’, and ‘age’, and the rest $15$ are DNA biomarkers. Including the identified important features and biomarkers in the stable DNN predictive model, the predicted MSPE reduces to $39.83$, indicating an additional nearly $62\%$ improvement, and the PCC increases to $0.637$, a further nearly $16\%$ improvement, compared with the corresponding counterpart from the PermFIT-DNN method. Intuitively, this result suggests that the stable DNN model performs better in the reduced feature space via the pre-screening step, more accurately identifying important features with the same training sample size, resulting in significantly improved prediction accuracy. The additional feature pre-screening procedure filters out a large proportion of uninformative noises and allows the subsequent feature importance test to be much more effective. Also, when comparing the important features and biomarkers identified by the PermFIT-DNN and HdFIT-DNN methods, we notice that $3$ clinical features (i.e. ‘more than $2$ sweetened snacks&beverages/day’, ‘lives with optimally fluoridated water’, and ‘age’) and $7$ DNA biomarkers are commonly claimed as important features by these two methods. The results from this real application further confirm the observations from the simulation study. Firstly, the stable DNN method demonstrates its robustness and effectiveness in modeling the complex association between features and disease outcomes, outperforming the RF method and leading to more accurate outcome predictions. Secondly, by incorporating the feature importance test procedure with the stable DNN model, critical features associated with disease outcomes can be effectively identified, resulting in a remarkable improvement in outcome prediction accuracy using the identified important features. Thirdly, the feature pre-screening procedure proves to be helpful, allowing the feature importance identification process to be more effective and further enhancing disease outcome prediction accuracy. Furthermore, it is worth noting that while the Lasso method can model high-dimensional association relationships with feature selection capability, it is not as robust as the PermFIT-DNN and HdFIT-DNN methods in identifying important features under complex association relationships. The stability and improved performance of PermFIT-DNN and HdFIT-DNN owing to the stable DNN for universally approximating complex functions accurately, especially in the high-dimensional setting, make them promising tools for analysing real-world data with numerous features and intricate relationships.

**Figure 3:**
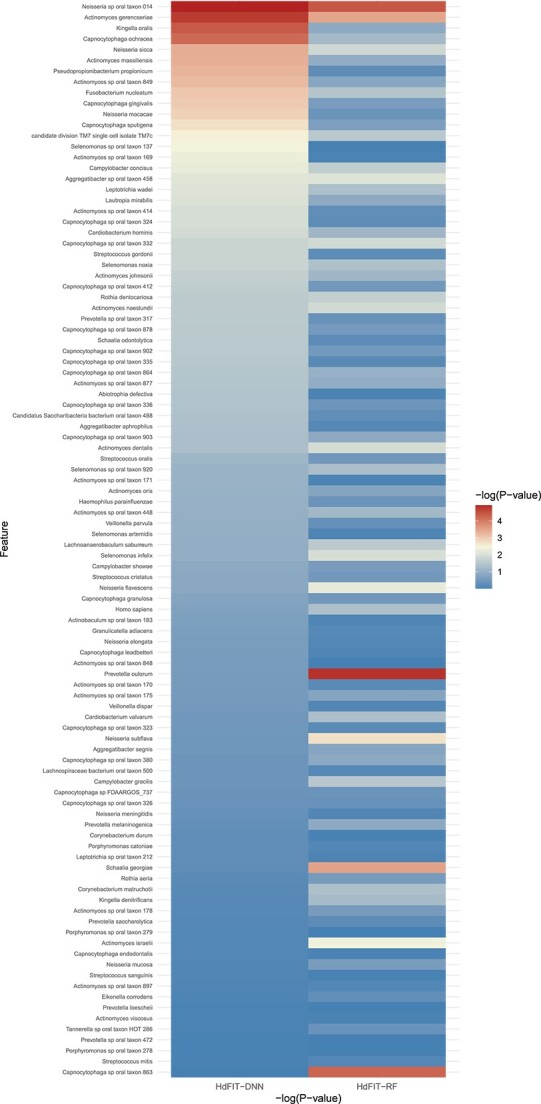
Feature importance heatmap.

Additionally, it is evident that HdFIT-DNN outperforms HdFIT-RF in all evaluation metrics considered for predicting the total caries lesions on testing samples. The biomarker feature importance heatmap determined by HdFIT-DNN and HdFIT-RF for the 100 prescreened biomarkers is presented in Fig. 3. Among the 100 biomarkers, HdFIT-DNN identified 15 important biomarkers. Most of these important biomarkers have been shown to be significantly associated with oral diseases in earlier studies. For example, Selenomonas sputigena has been shown to have a strong association and mechanistic roles with ECC [42]. *Capnocytophaga sputigena* was exclusively found in patients with initial lesions in enamel [43] and was associated with endotoxins in deep lesions in dentin [44]. Many studies found an association between Actinomyces gerencseriae and ECC [45–47], while many studies also identified an association between Neisseria sicca and ECC [48, 49].

On the other hand, HdFIT-RF identified 7 important biomarkers associated with total caries lesions. There are only two overlapping important biomarkers identified by both HdFIT-DNN and HdFIT-RF methods, i.e. Actinomyces gerencseriae and Neisseria sp. oral taxon. Among the two common biomarkers, we also noticed that HdFIT-DNN assigned substantially larger importance scores than HdFIT-RF. Additionally, using the important features identified by HdFIT-RF as input to the stable DNN for disease outcome prediction is less effective than using the important features identified by HdFIT-DNN as input to the stable DNN for outcome prediction. We attribute this to the stable DNN’s superiority in accurately approximating complex associations between biomarkers, clinical features, and disease outcomes. These findings highlight the potential of the stable DNN method, along with the feature importance test and pre-screening procedures, as powerful tools for analysing real-world data with high-dimensional features and complex relationships. Their ability to effectively identify critical features and improve outcome prediction accuracy can greatly contribute to advancing our understanding of disease mechanisms and facilitating more accurate disease outcome predictions in various clinical and research settings.

## Discussion

In this paper, we have introduced an analytical framework that enables the modeling of complex associations between behavioral factors, clinical features, high-dimensional molecular biomarkers, and disease outcomes while also conducting important feature identification based on robust statistical inference. Our findings emphasize that considering only behavioral factors and clinical features in a low-dimensional setting is insufficient for accurately characterizing diseases, leading to poor outcome prediction. Our analysis further demonstrates that molecular biomarkers can provide valuable information for predicting disease outcomes, but their intricate associations with disease phenotypes can be more effectively modeled by certain machine learning methods, such as the DNN model, resulting in significant improvements in prediction accuracy.

Additionally, our extensive numerical studies have shown that the PermFIT framework, in conjunction with the stable DNN machine learning model, can effectively identify important clinical features and biomarkers. By incorporating these essential features and biomarkers into the machine learning model, we can significantly enhance prediction performance. Moreover, in high-dimensional settings, a feature pre-screening step can further improve the performance of the PermFIT framework.

In comparison to PermFIT, our modified framework, named HdFIT, allows the machine learning models to more effectively identify important features with the same training sample size by constraining them to a more compact feature space. Importantly, the proposed HdFIT-DNN demonstrates effective identification of crucial features and considerable improvement in outcome prediction accuracy, even in situations where the feature space becomes very high, rendering the DNN and PermFIT-DNN approaches computationally intractable.

In comparison to the Lasso method, our HdFIT-DNN method achieves comparable prediction accuracy when the linear association assumption holds in high and medium high-dimensional settings. However, in cases where the linearity assumption does not hold, HdFIT-DNN significantly outperforms the Lasso method, showcasing its superiority in handling complex associations and high-dimensional data.

## Limitations

We acknowledge that in more complex scenarios, clinical outcomes may exhibit correlations due to genetic similarities such as twin data, repeated measurements, or exposure to similar environmental hazards, which could influence the performance of the HdFIT framework and may necessitate more robust methods. Further research is thus warranted to investigate its performance under such clinical scenarios and explore its suitability with different machine learning models. Additionally, though the high-dimensional feature screening presented is convenient and effective, it could be not robust enough to deal with more complicated high-dimensional profiles. More robust high-dimensional feature screening techniques should be developed.

**Key Points**

- **Integration of Informative Biomarkers**: combining low-dimensional behavioral and clinical features with informative high-dimensional molecular profiles can significantly improve disease outcome prediction and diagnosis.
- **Challenges in Integrating Complex High-dimensional Biomarkers**: due to the curse of high dimensionality and the complex interplay of behavioral and clinical features along with molecular biomarkers to influence disease outcomes, the integration of informative biomarkers can be highly challenging.
- **HdFIT Framework**: the HdFIT framework is introduced to address the challenges of integrating low-dimensional data with informative high-dimensional biomarkers by leveraging machine learning methods to model complex associations and evaluate each individual feature’s importa-nce with a robust statistical inference. HdFIT has consistently demonstrated its efficacy, interpretability and scalability, particularly when integrated with advanced machine learning models like the proposed stable deep neural networks (DNN), through comprehensive numerical studies and real data applications.

## Supplementary Material

Appendix_bbae709

## Data Availability

Deidentified screened and unscreened real data and the analytic R codes for duplicating the numerical analysis results are available on GitHub (https://github.com/BZou-lab/HdFIT).
